# Reconciling Pesticide Reduction with Economic and Environmental Sustainability in Arable Farming

**DOI:** 10.1371/journal.pone.0097922

**Published:** 2014-06-02

**Authors:** Martin Lechenet, Vincent Bretagnolle, Christian Bockstaller, François Boissinot, Marie-Sophie Petit, Sandrine Petit, Nicolas M. Munier-Jolain

**Affiliations:** 1 Institut National de la Recherche Agronomique, Unité Mixte de Recherche 1347 Agroécologie, Dijon, Côte d'Or, France; 2 Centre d'Etudes Biologiques de Chizé - Centre National de Recherche Scientifique, Beauvoir sur Niort, Deux-Sèvres, France; 3 Institut National de la Recherche Agronomique, Unité de Recherche 1121 Agronomie et Environnement, Colmar, Haut-Rhin, France; 4 Université de Lorraine, Vandœuvre-lès-Nancy, Meurthe-et-Moselle, France; 5 Chambre d'Agriculture des Pays de la Loire, Angers, Maine-et-Loire, France; 6 Chambre Régionale d'Agriculture de Bourgogne, Quetigny, Côte d'Or, France; Federal University of Viçosa, Brazil

## Abstract

Reducing pesticide use is one of the high-priority targets in the quest for a sustainable agriculture. Until now, most studies dealing with pesticide use reduction have compared a limited number of experimental prototypes. Here we assessed the sustainability of 48 arable cropping systems from two major agricultural regions of France, including conventional, integrated and organic systems, with a wide range of pesticide use intensities and management (crop rotation, soil tillage, cultivars, fertilization, etc.). We assessed cropping system sustainability using a set of economic, environmental and social indicators. We failed to detect any positive correlation between pesticide use intensity and both productivity (when organic farms were excluded) and profitability. In addition, there was no relationship between pesticide use and workload. We found that crop rotation diversity was higher in cropping systems with low pesticide use, which would support the important role of crop rotation diversity in integrated and organic strategies. In comparison to conventional systems, integrated strategies showed a decrease in the use of both pesticides and nitrogen fertilizers, they consumed less energy and were frequently more energy efficient. Integrated systems therefore appeared as the best compromise in sustainability trade-offs. Our results could be used to re-design current cropping systems, by promoting diversified crop rotations and the combination of a wide range of available techniques contributing to pest management.

## Introduction

Reconciling agricultural productivity with other components of sustainability remains one of the greatest challenges for agriculture [1]. A key issue will be to achieve substantial reductions in the level of pesticide use for environmental and health reasons [2], [3]. Agriculture in temperate climates is widely dominated by conventional intensive farming systems, with highly specialized crop productions and a heavy reliance on pesticides and mineral fertilizers [4]. However, increasing environmental concerns about intensive farming practices has contributed to the emergence of innovative farming systems, such as organic and integrated farming, typically presented as alternative paths to reduce pesticide use as compared to current conventional systems [5], [6], [7]. Whether these systems better meet sustainability criteria has been a matter of debate [8], [9]. Integrated farming, recently promoted in Europe through the 2009/128/EC European directive [10], is defined as a crop protection management based on Integrated Pest Management (IPM) principles, which emphasizes physical and biological regulation strategies to control pests while reducing the reliance on pesticides [11]. It can be regarded as an intermediate between conventional farming, with high levels of inputs, and organic farming, which prohibits the use of synthetic pesticides and fertilizers. Organic and integrated farming have in common the combined use of management approaches to replace, at least in part, synthetic inputs. However, unlike organic farming which is growing both in Europe (by 40 to 50% between 2003 and 2010 [12]) and in the US (by 270% between 2000 and 2008 [13]), integrated arable crop production is not expanding because it is perceived by farmers as a complex system which is difficult to implement, labour-consuming, and associated with reduced and unpredictable economic profitability [14], [15]. As a consequence, the amount of pesticides sprayed has only decreased slightly in Europe (−3.6% from 2000 to 2007 [16]) and in the US (−7.5% from 2000 to 2007 [17]). Moreover, this decrease can be partly attributed to the substitution of older chemistry, applied at high dosage, by new products that are efficient at lower doses, which actually cannot be considered as a reduction of pesticide reliance. In France, the national action plan, ECOPHYTO 2018, which had set a target of a 50% decrease in pesticide use by the year 2018, is currently far from achieving this goal [18].

**Figure 1:**
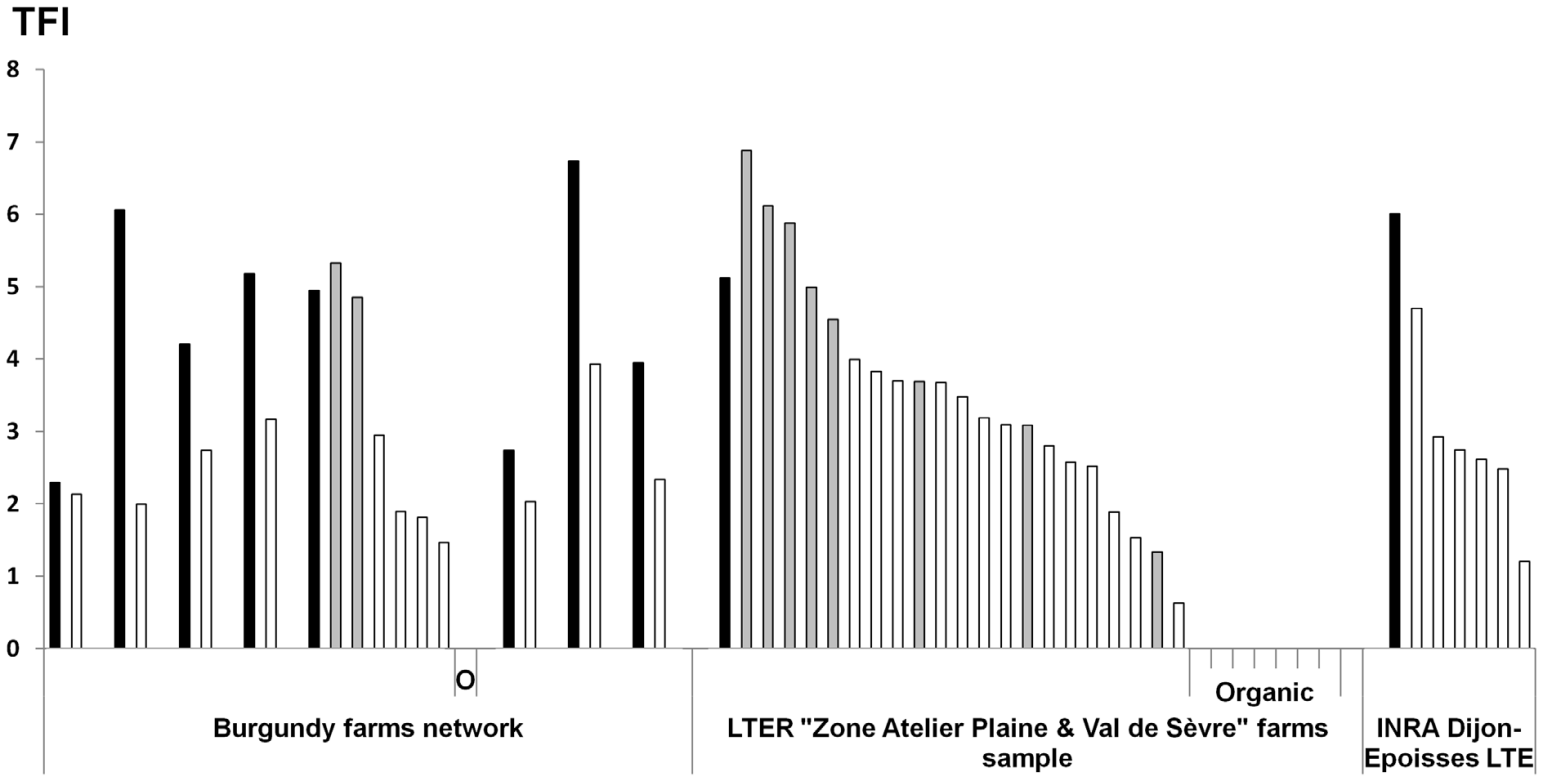
Distribution of the Treatment Frequency Index (TFI) for the studied arable cropping systems. Average TFI for each cropping system composing the study sample. At each site, black bars correspond to the local reference, grey bars to conventional cropping systems and white bars to integrated cropping systems. The sample also includes eight organic cropping systems with TFI = 0 and labelled with “O” or “Organic”. Details about the cropping systems are available in Dataset S1.

So far, assessments of cropping system sustainability have compared few – typically two or three – experimental prototypes that represent conventional, organic or integrated strategies [19], [20]. However, this approach fails to capture the diversity within each of these farming strategies. Given the diversity of crop management options within a conventional, an integrated or an organic strategy, which might lead to contrasted performances, the generic value of experimental results ignoring this variability may be argued. We assessed the sustainability of 48 cropping systems located in regions of intensive arable farming and covering a wide range of pesticide use levels and cultivation techniques such as crop rotations, from monoculture to highly diversified crop rotations, soil tillage (e.g. inversion tillage, shallow tillage or direct drilling), fertilization (mineral or organic fertilizers), or weed management (e.g. only based on herbicide use, including mechanical weeding). More details about the cropping system sample are available in the online SI section (Dataset S1). All the studied cropping systems were followed for between three and 12 years, between 1999 and 2012. Eight cropping systems were organic, 30 were based on integrated farming and 10 were conventional (Figure 1). Using eight sustainability indicators to evaluate the performance of the study systems, our aims were: (i) to identify possible conflicts between the reduction of pesticide reliance and other components of sustainability; and, (ii) to assess the potential of organic and integrated strategies for improving agricultural sustainability.

As the performance of a cropping system depends not only on the combination of management options it implements, but also on the local production situation [21] (including biophysical and socio-economic local aspects), we standardized the indicators of performance and pesticide use, using a ratio of the performances of the cropping systems over those of a local reference system. This enabled us to focus solely on the effects of the management strategies on sustainability indicators. The local references were cropping systems selected as representative of the most widespread crops and practices within each production situation. Pesticide use was measured as the Treatment Frequency Index (TFI), which is a commonly used indicator in Europe to estimate the cropping system dependence on pesticides [22]. In our sample, organic cropping systems did not use any pesticides (synthetic or natural) so their relative TFI, expressed as a ratio of the local reference TFI, was zero. Integrated cropping systems displayed TFI values that were on average half (−47%) of the local references (Table S1).

## Results

Table S1 presents the mean and standard deviation for each performance indicator according to the management strategy (organic, integrated and conventional). The second tab of Dataset S1 provides performance details for each cropping system of the sample.

### Productivity and energy efficiency

**Figure 2:**
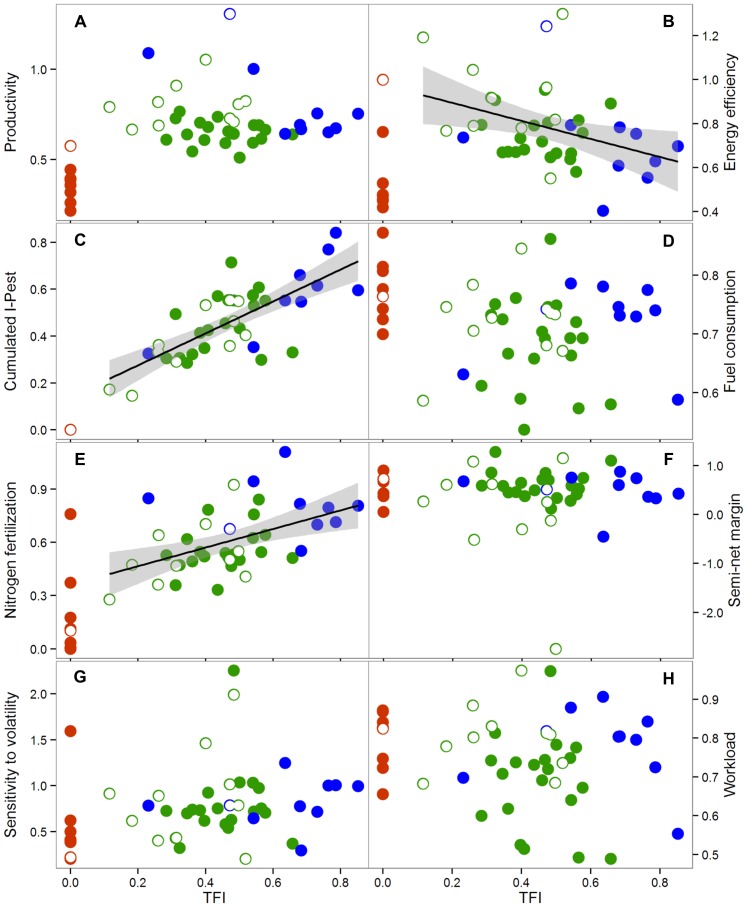
Relationship between sustainability indicators and relative TFI. Cropping system performances according to their relative TFI. Conventional, integrated and organic cropping systems are represented by blue, green and red symbols respectively. Filled symbols correspond to the cropping systems with grain crops only and empty symbols refer to the cropping systems including crops for which the whole above-ground biomass is harvested. Each sustainability indicators is expressed as the natural logarithm of the ratio between the cropping system and the local reference indicators. Linear regressions are represented with their standard error for cumulated I-Pest (Pearson correlation test: r_p_ = 0.74, P = 5*10^−8^), nitrogen fertilization (Pearson correlation test: r_p_ = 0.48, P = 0.002), and energy efficiency (Pearson correlation test: r_p_ = −0.38, P = 0.02). Performance metric included: a) productivity, b) energy efficiency, c) cumulated I-Pest, d) fuel consumption, e) nitrogen fertilization, f) semi-net margin, g) sensitivity to price volatility, h) workload.

**Figure 3:**
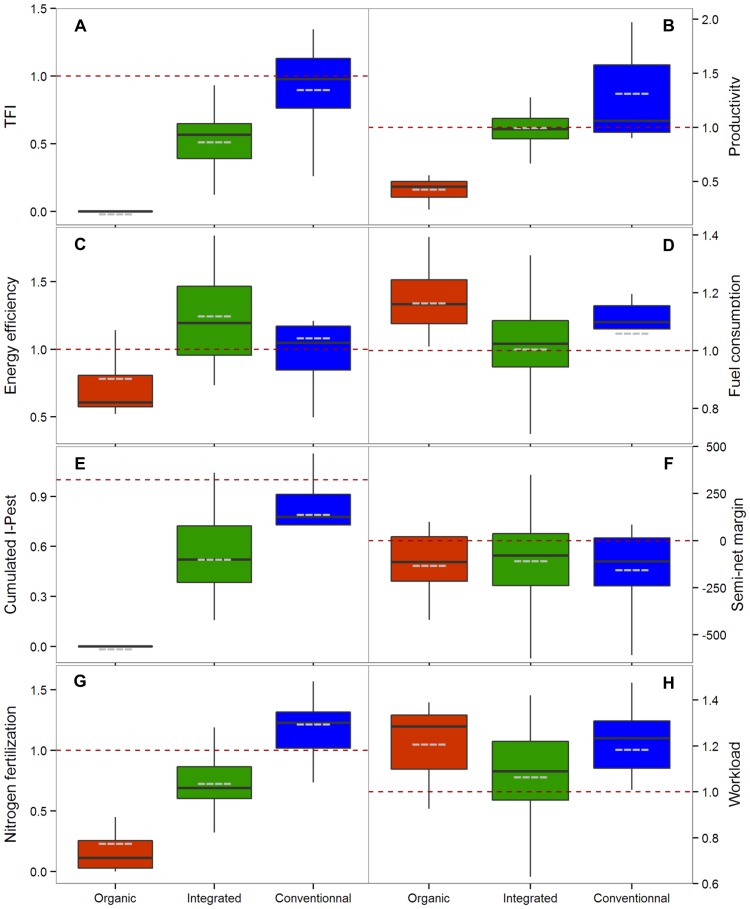
Cropping systems distribution according to sustainability indicators. Performance indicators are expressed as a ratio of the local reference indicator, except for semi-net margin, expressed as a difference with the local reference. Conventional, integrated and organic cropping systems are represented by blue, green and red box plots respectively. The horizontal black bars and grey dashed bars correspond to median and mean values respectively. The horizontal red dashed bar recalls the position of the local references. Outliers are not represented. Performance metrics included: a) Treatment Frequency Index, b) productivity (organic farming: one outlier, v = 0.78; integrated farming: two outliers, v1 = 1.48 and v2 = 1.87), c) energy efficiency (organic farming: one outlier, v = 1.72), d) fuel consumption (integrated farming: one outlier, v = 1.37; conventional farming: two outliers v1 = 0.8 and v2 = 0.88), e) cumulated I-Pest (conventional farming: two outliers v1 = 0.38 and v2 = 0.42), f) semi-net margin, g) nitrogen fertilization (organic farming: one outlier, v = 1.13; integrated farming: two outliers, v1 = 1.32 and v2 = 1.52), h) workload (conventional farming: one outlier v = 0.74).

Given the primary role of agriculture remains to produce food and other goods, we used an indicator of productivity, expressed as the total yearly amount of energy produced by a cropping system, whatever the crops cultivated (Figure 2a). The productivity of organic cropping systems was below that of their local reference (Figure 3b), ranging from −22% to −76%. For non-organic cropping system, productivity was uncorrelated to relative TFI (Figure 2a and Table 1), with some cropping systems that had a low reliance on pesticides even exceeding the productivity of the local reference. Cropping system productivity may strongly depend on crop type, especially if the whole above-ground biomass is harvested or not. Crops other than grain crops were frequently grown in integrated farming, as they are typically associated with low pesticide requirements and can contribute to weed control in subsequent crops [23]. They typically consist of forage crops, dedicated to livestock feeding with limited energy efficiency, or of crops used for non-food applications. However, distinguishing cropping systems based on grain crops or on crops in which all above-ground biomass is harvested did not change the observed pattern. In systems with grain crops only, productivity was not correlated with relative TFI (Table 1), suggesting that a reduction in pesticide use intensity may not be necessarily translated into a decrease in productivity. The second indicator of energy productivity we used was the energy efficiency of cropping systems, resulting from a ratio between energy output and energy input. It evaluated the ability of a cropping system to convert energy inputs into outputs. Organic cropping systems were significantly less energy efficient than other systems (Figures 2b and 3c, Table 2). Despite their energy consumption being lower (Table 2), notably due to their low reliance on nitrogen fertilizers, it was not sufficient to offset their limited productivity. Energy consumption was negatively correlated with relative TFI in integrated and conventional systems which cultivated only grain crops (Table 1). Energy efficiency was also negatively correlated with relative TFI in these systems, although the relationship was weak and only marginally significant (r_s_ = −0.35, P = 0.07). The systems with the highest energy efficiency, whether they included crops with all above-ground biomass harvested or not, were mostly integrated systems (Figures 2b and 3c).

**Table 1:** Rank correlation between TFI and sustainability indicators for integrated and conventional cropping systems.

**Spearman correlation**	**Productivity**	**Productivity (grain crops only)**	**Energy consumption**	**Energy consumption (grain crops only)**	**N fertilization**	**Pesticides environmental impact**
*r_s_*	*−0.17 (NS)*	*0.06 (NS)*	*0.30 (NS)*	0.42	0.51	0.67
*P-value*	*0.3*	*0.8*	*0.06*	0.03	9*10^−4^	4*10^−6^
**Spearman correlation**	**Fuel consumption**	**Energy efficiency**	**Semi-net margin**	**Sensitivity to prices volatility**	**Workload**	**Crop Sequence Indicator (Isc)**
*r_s_*	*0.06 (NS)*	−0.40	−*0.05 (NS)*	*0.22 (NS)*	9*10^−3^ (NS)	−0.22
*P-value*	*0.7*	0.01	*0.8*	*0.2*	*0.95*	0.2

Spearman rank correlation tests (α = 0.05). r_s_ is the Spearman correlation coefficient. Values of r_s_ followed by (NS) are not significant.

**Table 2:** Significantly different groups for a given performance indicator.

Indicator	Test designation	P-value	Statistic W
Productivity	Difference of productivity between organic cropping systems and the rest of the sample	2*10^−8^	318
Productivity	Difference of productivity between cropping systems including crops with the whole above-ground biomass harvested and the rest of the sample	5*10^−4^	76
Energy efficiency	Difference of energy efficiency between organic cropping systems and the rest of the sample	0.005	258
Energy consumption	Difference of energy consumption between organic cropping systems and the rest of the sample	0.001	272
Fuel consumption	Difference of fuel consumption between organic cropping systems and the rest of the sample	0.02	72
N fertilization rate	Difference of N fertilization rate between organic cropping systems and the rest of the sample	2*10^−4^	285
Sensitivity to price volatility	Difference of sensitivity to price volatility between organic cropping systems and the rest of the sample	0.01	250
Workload	Difference of workload between cropping systems based on organic fertilization and the rest of the sample	0.045	189
Crop sequence indicator	Difference of Isc between organic cropping systems and conventional cropping systems	0.01	69
Crop sequence indicator	Difference of Isc between integrated cropping systems and conventional cropping systems	0.001	255
Crop sequence indicator	Difference of Isc between cropping systems including legumes and the rest of the sample	6*10^−6^	63

Mann-Whitney tests (α = 0.05). All P-values are below 0.05, indicating that the differences between means of the sub-samples are significant for the corresponding indicators.

### Environmental impact

The environmental impact of cropping systems and their reliance on external inputs were assessed with the indicator I-Pest [24] and with estimates of fuel and nitrogen fertilizer consumption. I-Pest is a predictive indicator that assesses the environmental impacts of pesticide use as the risk of contamination of the air, and surface and ground waters (see Figure S1). As the organic cropping systems composing the sample did not used synthetic or natural pesticide, their cumulated I-Pest was 0. As expected for the rest of the sample, cumulated I-Pest was strongly and positively correlated to relative TFI (Figure 2c and Table 1). Fuel and nitrogen fertilizers together amounted to more than 60% of the total energy inputs for all tested cropping systems. Organic systems consumed more fuel than the rest of the sample (Table 2), with their average consumption exceeding the local references by 17% (Figure 3d). Organic cropping systems had, nonetheless, a lower reliance on N fertilization than the rest of the sample (Figure 3g, Table 2), in line with their lower yield targets and the frequent occurrence of crops with low N requirements used in organic rotations. No relation was detected between fuel consumption and relative TFI in non-organic systems (Figure 2d, Table 1), but a positive correlation was clearly visible between relative TFI and the amount of nitrogen fertilizers applied (Figure 2e).

### Economic sustainability and workload

Economic sustainability was assessed by considering (i) the profitability, i.e. the average semi-net margin over a range of ten real price scenarios for agricultural products, fuel and fertilisers, and (ii) the sensitivity of this profitability in a context of price volatility, i.e. the relative standard deviation of the semi-net margin. The range of price scenarios used for the calculations was set to reflect the variability of the economic context over the last decade. Profitability, when averaged over the ten price scenarios was not correlated with relative TFI for integrated and conventional systems (Figure 2f and Table 1), and no significant difference appeared with organic systems (Mann-Whitney test, P>0.9). It suggests that low pesticide use would not necessarily result in lower economic return. The strong variability observed within each class (Figure 3f), most notably for integrated cropping systems, confirmed that strategies to reduce pesticide use could even lead to an increase in profitability. As integrated cropping systems were, in contrast to organic systems, evaluated with a conventional price reference, the most profitable integrated systems were able to efficiently reduce their production costs. No relation was detected between the sensitivity to price volatility and relative TFI in conventional and integrated systems (Figure 2g, Table 1). Sensitivity to price volatility was significantly lower in organic cropping systems than in other systems (Table 2), most probably because: (i) they were based on more diversified crop rotations, which spread risks and buffered semi-net margin at the farming system scale; and, (ii) their crop rotations typically included crops with low N demand, that had reduced reliance on N inputs, whose price is directly related to the volatile price of fossil fuels.

The issue of social sustainability was addressed using the ‘workload’ indicator, which gives emphasis to the potential for bottlenecks where available workforce is a limiting factor at the farm scale (Figure 2h). Workload was calculated for each technical operation but excluded time devoted to transport and crop monitoring. Workload was found not correlated with relative TFI in non-organic cropping systems (Table 1), and no significant difference was found with the organic group (Mann-Whitney test, P>0.1), so that reducing pesticide use does not necessarily imply an increased workload. Indeed, in integrated systems, labour requirements ranged from low to high relative values (Fig 2h). The level of workload was, however, related to the type of fertilization, with cropping systems having organic fertilization requiring an average of 13% greater working time, as compared to mineral fertilizer-based cropping systems (Table 2).

### Crop diversity

Diversification of crop rotations is often presented as an efficient management tool for controlling pests and to improve agricultural sustainability [25], [26]. We used a crop sequence indicator, Isc [27], which estimates the consistency of the crop sequence with regard to the potential of input reduction, by addressing effects of crop rotation on pathogens, pests, weeds, soil structure and nitrogen supply of preceding crops. Even if no significant correlation appeared between Isc and relative TFI (Table 3), organic and integrated cropping systems displayed significantly higher Isc values than conventional systems (Table 2). A negative correlation between Isc and productivity suggests that diversifying crop rotation may reduce cropping system productivity (Table 3), but the Spearman correlation test was no longer significant when organic cropping systems were excluded (P = 0.07). We did not detect any significant relationship between energy efficiency and crop diversification, whether organic cropping systems were included or not (P = 0.44). No correlation was observed between Isc and semi-net margin, but workload appeared to be lower for systems with higher Isc (Table 3). We found the expected negative correlation between Isc and N fertilization rates, and consequently between Isc and energy consumption (Table 3). We focused therefore more particularly on cropping systems including legume crops, which also displayed higher Isc values than the rest of the sample (Table 2). The role of legume in improving energy efficiency at the cropping system scale was clearly demonstrated by the correlation between the frequency of occurrence of legumes in the crop rotation and the energy efficiency (r_s_ = 0.37, P<0.05). The sensitivity to price volatility was negatively correlated with the frequency of occurrence of legumes in the crop rotation (r_s_ = −0.33, P = 0.02), but positively correlated with the level of N fertilization (r_s_ = 0.49, P = 5*10^−4^). Fostering exogenous N independence therefore appeared as an efficient way to limit income variability.

**Table 3:** Rank correlation between Crop Sequence Indicator Isc and sustainability indicators.

**Spearman correlation**	**Productivity**	**Energy consumption**	**N fertilization**	**Pesticides environmental impact**	**Fuel consumption**
*r_s_*	−0.35	−0.42	−0.41	−0.39	−*0.07 (NS)*
*P-value*	0.01	0.003	0.004	0.006	*0.7*
**Spearman correlation**	**Energy efficiency**	**Semi-net margin**	**Sensitivity to price volatility**	**Workload**	
*r_s_*	−*0.05 (NS)*	*0.10 (NS)*	−*0.23 (NS)*	−0.29	
*P-value*	*0.7*	*0.5*	*0.1*	0.04	

Spearman correlation tests (α = 0.05). r_s_ is the Spearman correlation coefficient. Values of r_s_ followed by (NS) are not significant.

## Discussion

This work was aimed at detecting cropping systems able to reconcile low pesticide use and other components of sustainability. Our original multiple dimensions approach, based on a precise description of management practices, was designed to compare and contrast numerous cropping systems from different production situations. This approach, applied at the large-scale, was able to provide generic knowledge about potential trade-offs between the different issues of agricultural sustainability.

### Sustainability of integrated and organic farming

Our results show that achieving a low level of pesticide use is possible without triggering negative side effect on any of the components of cropping system sustainability we assessed in this study. Integrated cropping systems were not only associated with low pesticide use and low risks for contamination of air and water with pesticide residues; they also displayed lower energy consumption than more intensive cropping systems and are likely to improve energy efficiency without impact on productivity and profitability. Lower pesticide usage in arable cropping systems did not imply a heavier workload, another critical point conditioning strongly the adoption of an innovative strategy.

Organic farming prohibits the use of synthetic pesticides and fertilizers, and this approach is often associated with low nitrogen fertilization, as observed in our sample. In addition to the positive effects on environmental quality, numerous studies underlined other environmental benefits of organic farming such as effects on pollinator dynamics [28], on landscape floristic composition [29], as well as on soil microbial diversity [5], [30]. Here we demonstrated that organic farming does not necessarily affect profitability and workload, and conversely, might strengthen farm financial stability in a variable and unpredictable economic context. Organic cropping systems were however less productive and less energy efficient than integrated systems in our sample. Although highly dependent on crops and production context, productivity in organic farming was already reported as lower than in conventional farming by other comparative studies [31]. The poor land use efficiency associated with organic farming is a key issue in the current land sharing – land sparing debate about the growing competition for land use [32], and notably urban sprawl [33] as well as the necessity to keep natural spaces undisturbed [34], [35]. Both aspects – environmental benefits of organic farming and the limited productivity per unit of land – should therefore be considered by decision makers in their incentives for sustainable agriculture.

### Crop diversification

Our results support the hypothesis that crop diversification may be an effective means to enhance cropping system performance. At the cropping system scale, crop diversification provides agronomic advantages, such as the regulation of pests, diseases and weeds [36], [37], [25]. In our sample, the most diversified cropping systems, which displayed the highest values of the crop sequence indicator, Isc, were indeed less dependent on pesticides. Their low environmental impact on water and air quality makes crop diversification an interesting potential pathway for reducing the damage caused by agriculture on natural resources (e.g., biodiversity [38]), as well as on human health (e.g. neurological degenerative disorders [39]). By mitigating the adverse effects of climate variability, crop diversification may also improve system resilience for productivity [40], with the increasing likelihood of extreme weather events requiring farm adaptation [41]. Economic market volatility is an additional source of variation and risk factor for farm economic stability. We found that crop diversification, particularly through the introduction of legumes in the crop rotation, is likely to limit dependence on inputs that have unstable prices. By allowing a decrease in the use of exogenous N fertilizer across a crop rotation, legume cultivation reduces production cost fluctuations and consequently makes the cropping system less sensitive to market volatility. Legumes come with a supplementary advantage [42] in the face of the considerable amount of fossil energy necessary to produce mineral N fertilizers, and we noted a substantial increase in energy efficiency for crop rotations where legume crops are more frequent. The most part of legumes introduced as diversification crops are however forage crops, and livestock production is commonly considered more energy consuming than plant production [43]. Conversely, the use of farmyard manure may contribute to reduce mineral fertilizers reliance for grain and forage crop production. The necessity of (i) integrating these situation-dependent parameters into energy balancing calculations, and, (ii) evaluating other environmental indicators [44] will be critical for the assessment of livestock production as a management option for enhancing agricultural sustainability.

A key agronomical advantage of crop diversification is related to the management of weed resistance to herbicides. Crop diversification is an efficient means to alternate herbicide modes of action and to introduce diversified measures of weed control, allowing changing selection pressure on weed communities and thus maintaining a sensitive weed population (i.e. maintaining high herbicide efficiency) [45].

Our results demonstrate a negative correlation between the Isc value and workload. We can nevertheless assume that diversifying crop rotations increases cropping system complexity and time devoted to field observations. Another aspect is that crop diversification may lead to a more evenly distributed workload over the seasons. Crops diversity implies a greater diversity in sowing and harvest periods, which are both times of peak labour that strongly influence task organisation and farmer decision making [15]. By reducing the amplitude of these peaks in labour, crop diversification could contribute to ensuring greater farmer decisional flexibility at the farm scale.

Beyond technical and organizational issues at the farm level, diversifying crop production as a component of an integrated strategy at regional or national scale would inevitably lead to important changes in production volumes, as well as markedly changing agricultural sectors within each production basin. It would definitely require an adaptation in the organisation of the whole agricultural sector and the development of new local markets. These economic and social lock-ins are rightly highlighted as the main limiting constraints hindering crop diversification [46]. However, by creating a particular economic sub-context, niche markets can be attractive and able to support innovation. Promoting such niche markets, for integrated farming development, would be the first step along an accelerating cycle of improvement based on mutually positive feed-backs between production and outlets.

## Materials and Methods

In all cases, the field studies did not involve endangered or protected species.

For future permissions about the private farm network of Burgundy, please contact Marie-Sophie Petit (co-author of the research article, Chambre Régionale d'Agriculture de Bourgogne) and Sandrine Petit (co-author of the research article, INRA).

For future permissions about the private farms survey carried out on the LTER “Zone Atelier Plaine & Val de Sèvre”, please contact Nicolas Munier-Jolain (corresponding author, INRA).

### Study areas

The main objective of this study was to highlight potential conflicts between pesticide use and a set of sustainability indicators, so the cropping systems we consider were selected to maximize the contrast across the range of possible pesticide use intensities. The sample of cropping systems we used originates from:

- A long term experiment conducted since 2000 at the INRA Dijon-Epoisses farm in Bretenière (Burgundy, eastern France; 47°20′N, 5°2′E) in order to assess Integrated Weed Management-based cropping systems [15], [26]. Seven cropping systems were tested between 2000 and 2012 including different combinations of technical levers likely to reduce pesticide reliance.
- An experimental network (bringing together 14 cropping systems) monitored (1) by the local agricultural extension services and coordinated by the Chambre Régionale d'Agriculture de Bourgogne, and (2) by the INRA de Dijon. This network involved contrasting private farms of the Burgundy region, and was developed to test feasibility of innovative cropping systems with reduced pesticide use in a realistic context.
- A survey of private farms carried out in 2010 on the LTER “Zone Atelier Plaine & Val de Sèvre” [47] located in the Poitou-Charentes region (450 km^2^ study area in western France), and set up to explore a diversity of pesticide reliance, including organic farming, conventional intensive systems, and intermediate, IPM-based systems. Twenty nine varied cropping systems were surveyed in this area.

### Cropping systems classification

Details of cropping systems, including crop sequences, performances, and detailed crop management operations are made available in the Dataset S1 and S2. Cropping systems were considered as conventional, integrated or organic according to the following rules. Cropping systems complying with the organic farming specifications were treated as ‘organic’. Other systems were considered as ‘integrated’, when they were based either on diversified crop rotations including unusual alternative crops for the production situation (i.e. not present in the local reference crop rotation), or when crop management included at least one non-chemical management approach that contributed to the control of pests, diseases or weeds. These included for instance biocontrol, mechanical weeding and false seed bed techniques. Systems that were not classified as ‘organic’ or ‘integrated’ were classified as ‘conventional’.

### Local reference definition

For each of the 48 systems, a local cropping system reference was selected to reflect the most widespread crop rotation and associated technical management, as well as the typical agricultural performance in the production situation. Using this local cropping system reference made it possible to distinguish the effects of agronomic strategies from the effects of the production situation (soil, climate, economic and social context) when assessing the various components of sustainability of each cropping system. The Dijon-Epoisses experiment included a reference standard system that follows recommendations of local extension services [26], and which was used as the local reference. For each farm of the network across Burgundy, the local reference was defined as the cropping system implemented within the farm before the set-up of the alternative cropping system, even though crop management sequences were slightly updated according to expert appraisal to match with current standards (e.g. active ingredients allowed). For the Zone Atelier “Plaine et Val de Sèvre”, local expert knowledge was used to select one system from the survey, with a standard crop rotation for the area, and a crop management representative of local practices. This system was then used as the local reference for all the remaining surveyed systems of the area.

### Assessment of sustainability

The assessment of sustainability at the cropping system scale was based on a range of indicators covering economic, environmental and social issues. The Treatment Frequency Index (TFI) [22] estimates the number of registered doses applied, for each pesticide, per hectare and per crop season. Averaged over the cropping system, this indicator summarizes the level of dependence on pesticides, which should be distinguished from the environmental impact of pesticide use. This indicator is calculated for each pesticide application according to the following formula:
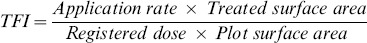



The application rate and the registered dose were both expressed for a given commercial product (which possibly contains several active ingredients). The recommended application dose depends obviously on the treated crop and on the targeted pest. Here we defined the registered dose as the lowest application dose which is recommended for a given crop. The TFI for a given crop season was then calculated as the sum of the TFI for each pesticide application performed during this crop season. Productivity was evaluated as the amount of energy harvested yearly. This approach allowed the comparison of different crop rotations that included crops with different yielding potentials and different energy content. For each crop, yields were transformed into the energy metric using their Lower Heating Value (LHV) [48], which corresponds to the amount of energy released per unit of mass by the combustion of the harvested biomass. Energy consumption was estimated from the conversion of inputs into energy according to the Dia'terre reference database [48]. Dia'terre is an assessment tool developed by the Agency for the Environment and Energy Management (ADEME) in the framework of the French Plan for Energy Performance (PPE) to evaluate a carbon-energy balance at the farm scale. The reference database used to design this assessment tool provides energy values for indirect energy consumption associated with the production of farming inputs. For instance, the calculation of the energy cost associated with the production of nitrogen fertilizers integrates the energy necessary from raw material production (e.g. Haber–Bosch process) through materials processing, manufacture and distribution. We used the reference energy cost provided by the carbon calculator Dia'terre to compute the energy balances of the cropping systems. In this way, the inputs necessary for crop production were converted into energy, using the energy cost of fertilizers, pesticides, seeds, water spread for irrigation, fuel consumed by the equipment and the amount of steel necessary to manufacture this equipment, i.e. energy cost of mechanization (see Dataset S3). The energy requirements for preparing farmyard manure are farm-specific and very difficult to quantify precisely. A simplification was consequently required: following previous studies based on energy balancing methods in crop production [49], the energy equivalent of farmyard manure was equated with that of the mineral fertilizers they substituted (using a substitution value related to the fertilizing efficiency of manure). Energy efficiency was computed from the ratio between productivity and energy consumption. For assessing the economic productivity, the gross product derived from the direct conversion of crops yields into economic values. The ‘semi-net’ margin was calculated as the gross product per hectare from which we subtracted the input costs (fertilizers, pesticides, seeds, fuel, water and mechanisation). This ‘semi-net’ margin assessed the system profitability without taking into account subsidies or incentives. The sensitivity to price volatility was defined as the relative standard deviation of the semi-net margin calculated over ten contrasting real price scenarios selected between 2000 and 2010, and thus measured the ability of a cropping system to generate a stable income in a variable economic context. The ten scenarios integrated the prices of crops but also the prices of volatile inputs such as fertilizers or fuel. Each price scenario was defined at a given moment between 2000 and 2010, and it therefore reflected the correlations between the prices of crop products and inputs. This approach notably made it possible to integrate better the effects coming from crop diversity (proportion of cereal crops, oil crops or protein crops) on cropping system profitability and economic stability. Fuel consumption and workload were estimated according to in-field cropping operations only, without considering fuel and time consumed for farm-to-field transports, or extra-workload dedicated to equipment maintenance or field observations. The size, fuel requirements and working output of the various equipment types were standardized for all cropping systems, and defined from a national database [50], consistent with the aim of evaluating management strategies, and of ignoring the potential effects of the equipment specifications (See Dataset S4 for the details of the equipment used for the calculations).

Pesticide environmental impact was expressed as cumulated I-Pest [24]. This indicator measures the risk associated with pesticide application for three compartments of the environment, namely the air, the surface water and the groundwater. This risk indicator, ranging from 0 to 1 (maximum risk), and calculated for each active substance application, is based on: (i) field inherent sensitivity to pesticide transfer toward these three compartments; (ii) characteristics of the active substance (e.g. ecotoxicity, mobility, half-life); and, (iii) information about the conditions of the spraying operation (e.g. amount of active substances employed, canopy cover at the date of treatment) in order to calculate three impact factors, one for each compartment. I-Pest index is obtained using fuzzy decision trees that allow the aggregation of these three impact factors into one synthetic indicator. The diagram presented in Figure S1 illustrates how this indicator of pesticide environmental impact was computed for each pesticide active substance that was sprayed within the field.

The crop sequence indicator Isc [27] is used as an additional indicator to quantify the agronomic effects of crop diversification. Isc ranges on a qualitative scale between 0 and 10 (best value) and is calculated as shown in the following equation:
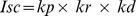



Isc is based on the assessment of the effects of the previous crop on the current crop (kp), with respect to the development of pathogens, pests and weeds, to soil structure and nitrogen supply. kp, ranging between 1 and 6, was assessed for 470 couples crop/previous crop. kp is corrected by two factors taking into account the crop frequency (kr ranging between 0.3 and 1.2) and the crop rotation whole diversity (kd ranging between 1.0 and 1.4). Isc yields respectively 0.5 for wheat monoculture, 3.3 for a rape/wheat rotation, 5.1 for a rape/wheat/barley rotation, and 7.6 for a maize/wheat/sunflower/spring barley rotation.

### Computation of sustainability indicators at the cropping system level

As a first step each indicator was calculated for each cropping operation composing our database (Dataset S2). These values of indicators were summed over the crop season year, and then averaged across years and across plots, each plot being considered as a replicate of a given cropping system. Each indicator was therefore calculated at the cropping system level, integrating (i) the different crops composing the crop sequence, (ii) the variability of crop production related with the inter-annual climatic variability, and (iii) the possible variation in plot properties.

All sustainability indicators were expressed per hectare and per year. For distinguishing specifically the effects of the management strategy on cropping system sustainability from the effects of the production situation, each indicator computed for a given cropping system was then expressed as a ratio (or as a distance in the case of semi-net margin) between the system indicator and the local reference indicator. To increase the quality of the graphs drawing the relationship between sustainability indicators and pesticide use, values of assessment indicators were translated into natural logarithm (Figure 2), which reduced the visual effect of extreme values.

### Statistical analyses

Spearman and Pearson correlations were estimated using the ‘rcorr’ correlation matrix function in the *Hmisc* package of R v2.15.0 [51]. The difference between the means of two sub-samples for a given indicator was tested with a non-parametric Mann-Whitney test (‘wilcox.test’ function with two samples) in the *stats* package of R v2.15.0.

## Supporting Information

Figure S1 Simplified description of the assessment process of pesticide environmental impact in the I-Pest model.
(TIF)

Dataset S1 Cropping systems details. A.xlsx file describing the cropping systems of the studied sample (e.g. crop rotation, tillage and weed management strategies). This file also provides information about the local reference associated with the evaluation of each cropping system. The second tab provides the respective performances of each cropping system described in the first tab.
(ZIP)

Dataset S2 Cropping operations database. A.xlsx file which provides the details of all cropping operations carried out in each cropping system: type of cropping operation, date (when recorded), application rates (for pesticides, fertilizers, seeds and irrigation) and proportion of the plot surface targeted.
(ZIP)

Dataset S3 Energy balancing database. A.xlsx file with two sheets. The first sheet provides energy cost values for inputs: pesticides active substances, fuel, fertilizers, irrigation water and seeds. The second sheet includes the Lower Heating Values (LHV) for usual crops, that is to say the energy contained in one mass unit of crop harvested.
(RAR)

Dataset S4 Standard equipment characteristics. A.xlsx file describing the technical characteristics of the standard equipment we associated with each cropping operation. Details include the purchase price, the payback period and the maintenance cost to calculate the mechanization costs, but also the equipment size and weight, the working output, the fuel consumption rate and the energy cost value.
(RAR)

Table S1 Means and standard deviations for the range of performance indicators according to the management strategy. A.xlsx file summarizing and comparing the performances of organic, integrated and conventional cropping systems which compose the study sample. Significant difference between groups was tested with a Mann-Whitney test.
(ZIP)
