# Supplementary figures and images for: Reconciling Pesticide Reduction with Economic and Environmental Sustainability in Arable Farming
Source: PLoS One. 2014 Jun 2;9(6):e97922. doi: 10.1371/journal.pone.0097922 (PMC4041714; doi:10.1371/journal.pone.0097922)

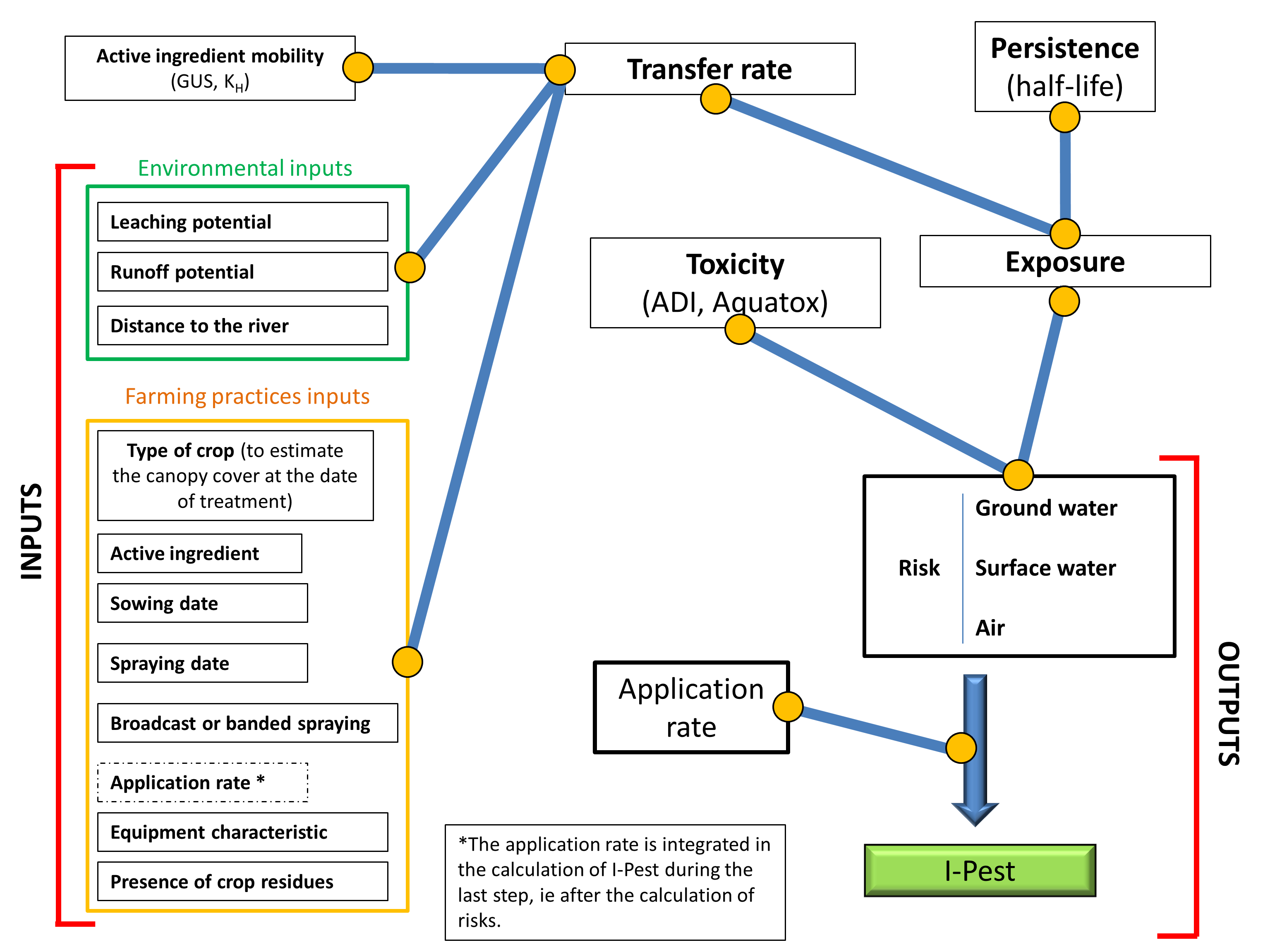

Supplement: Figure S1 — Simplified description of the assessment process of pesticide environmental impact in the I-Pest model. (TIF) [file pone.0097922.s001.tif]
